# Reversal of edoxaban-induced anticoagulation by the four-factor prothrombin complex concentrate Beriplex^® ^in a rabbit model

**DOI:** 10.1186/cc13288

**Published:** 2014-03-17

**Authors:** E Herzog, F Kaspereit, W Krege, B Doerr, J Mueller-Cohrs, I Pragst, Y Morishima, G Dickneite

**Affiliations:** 1 CSL Behring GmbH, Marburg, Germany; 2 Daiichi Sankyo Co., Ltd, Tokyo

## Introduction

The oral direct and selective factor Xa inhibitor edoxaban (Daiichi Sankyo) is currently available in Japan for the prophylaxis of venous thromboembolism (VTE) in patients undergoing major orthopedic surgery and is undergoing investigation in phase III trials for the prevention of stroke in patients with atrial fibrillation and the treatment and secondary prevention of VTE. The primary complication of any available anticoagulant therapy is the risk of bleeding. Rapid reversal of anticoagulation may be necessary in patients requiring emergency treatment due to uncontrolled bleeding. Prothrombin complex concentrates (PCC) are frequently used to reverse the effect of vitamin K antagonists such as warfarin and have also been suggested to be potentially effective in reversing the effects of the new oral anticoagulants. The present study was therefore designed to determine whether the four-factor PCC Beriplex^® ^can effectively reverse bleeding and normalize coagulation following edoxaban administration in a rabbit kidney injury model.

## Methods

Rabbits were treated with a high intravenous bolus dose of edoxaban (1,200 μg/kg) followed by the administration of Beriplex^® ^(25 to 75 IU/kg). Bleeding was assessed based on the time to hemostasis and the total blood loss after induction of a standardized kidney injury. In parallel, the following biomarkers of hemostasis were determined: factor Xa inhibition, prothrombin time (PT), activated partial thromboplastin time (aPTT), whole blood clotting time (WBCT), and thrombin generation (TGA).

## Results

The results confirmed increased and prolonged bleeding of edoxaban-treated animals following standardized kidney injury compared with vehicle administration. Parallel monitoring of biomarkers of hemostasis showed a prolongation of PT, aPTT, WBCT, and changes in thrombin generation parameters. Subsequent administration of Beriplex^® ^resulted in a dose-dependent reversal of edoxaban-induced bleeding as indicated by reduced time to hemostasis and total blood loss. Both parameters achieved statistical significance compared with placebo at the Beriplex^® ^dose of 50 IU/kg under fully blinded study conditions. The biomarkers correlating best with Beriplex^®^-mediated edoxaban anticoagulation reversal included PT, WBCT and endogenous thrombin potential.

## Conclusion

In summary, Beriplex^® ^treatment effectively reversed edoxaban-induced anticoagulation in an animal model of acute bleeding at clinically relevant dose levels.

